# Quantitative measurement of cortical superficial siderosis in cerebral amyloid angiopathy

**DOI:** 10.1016/j.nicl.2023.103447

**Published:** 2023-06-01

**Authors:** T.W. van Harten, E.A. Koemans, S. Voigt, I. Rasing, M.J.P. van Osch, M.A.A. van Walderveen, M.J.H. Wermer

**Affiliations:** a Department of Radiology, Leiden University Medical Center, Leiden, The Netherlands; b Department of Neurology, Leiden University Medical Center, Leiden, The Netherlands

**Keywords:** Cortical superficial siderosis, Segmentation, Quantification

## Abstract

Cerebral amyloid angiopathy (CAA) is a cerebrovascular disease affecting the small arteries in the brain with hallmark depositions of amyloid-β in the vessel wall, leading to cognitive decline and intracerebral hemorrhage (ICH). An emerging MRI marker for CAA is cortical superficial siderosis (cSS) as it is strongly related to the risk of (recurrent) ICH. Current assessment of cSS is mainly done on T2*- weighted MRI using a qualitative score consisting of 5 categories of severity which is hampered by ceiling effects. Therefore, the need for a more quantitative measurement is warranted to better map disease progression for prognosis and future therapeutic trials. We propose a semi-automated method to quantify cSS burden on MRI and investigated it in 20 patients with CAA and cSS. The method showed excellent inter-observer (Pearson’s 0.991, P < 0.001) and intra-observer reproducibility (ICC 0.995, P < 0.001). Furthermore, in the highest category of the multifocality scale a large spread in the quantitative score is observed, demonstrating the ceiling effect in the traditional score. We observed a quantitative increase in cSS volume in two of the 5 patients who had a 1 year follow up, while the traditional qualitative method failed to identify an increase because these patients were already in the highest category. The proposed method could therefore potentially be a better way of tracking progression.

In conclusion, semi-automated segmenting and quantifying cSS is feasible and repeatable and may be used for further studies in CAA cohorts.

## Highlights

• A semi-automatic segmentation and quantification of cSS on SWI-MRI.
• This segmentation and quantification of cSS is feasible and reproducible.
• Quantification of cSS may be used as a more continuous variable of cSS severity.

## 1 Introduction

Cerebral amyloid angiopathy (CAA) is a neurovascular disease characterized by progressive accumulation of the protein amyloid β in the walls of small cortical and leptomeningeal vessels. CAA is a common cause of lobar intracerebral hemorrhage (ICH) and cognitive decline in the elderly (Vinters, 1987, Charidimou et al., 2017, Jäkel et al., 2022). A diagnosis of probable or possible CAA can be established with the Boston criteria which are mainly based on clinical symptoms together with hemorrhagic and white matter MRI markers (Knudsen et al., 2001, Charidimou et al., 2022). An important MRI marker of CAA is cortical superficial siderosis (cSS) (Linn et al., 2010, Wermer and Greenberg, 2018). Although the cause of cSS is not entirely clear, the prevailing hypothesis include previous acute convexity subarachnoid hemorrhage leading to hemosiderin deposited in the subpial space (Linn et al., 2010). On T2(*) or susceptibility weighted MRI, cSS is visible as a hypointense band following the outer layer of the cortex. Presence, extent and progression of cSS on MRI are all associated with increased risk of future ICH (Charidimou et al., 2017, Greenberg and Smith, 2019, Greenberg et al., 1993, Charidimou et al., 2019), and are considered important prognostic markers in clinical practice.

For scoring the severity of cSS, the cSS multifocality rating scale has been developed and this score has extensively been employed to evaluate and monitor extent and progression of cSS. In this scale, each hemisphere is scored separately for cSS and subsequently the scores of both hemispheres are added to yield a total multifocality score ranging from 0 to 4. Although the multifocality rating scale has proven to be an important predictor of future ICH risk over its entire range (Charidimou et al., 2017), it cannot be used to monitor future progression of cSS in patients who are already categorized as having severe and multifocal cSS (ceiling effect). Also, subtle increases in cSS within sulci that are already affected by cSS will not be reflected in the multifocality scale.

A more sensitive and quantitative measure of cSS is therefore much needed. A score reflecting the total cortical area affected by cSS seems a logical extension of the visual score that would not suffer from the drawbacks of a categorical score. The aim of the current study is to develop a semi-automatic tool for the quantification of cSS in patients with CAA.

## 2 Materials and methods

For this study we used MRI data from patients with Dutch-type hereditary CAA (D-CAA) and sporadic CAA (sCAA) who participated in two ongoing longitudinal natural history studies (FOCAS and AURORA) in the Leiden University Medical Center (LUMC, Leiden, The Netherlands). MRI data from 20 patients (15 D-CAA and 5 sCAA) with a varying range of cSS severity on the multifocality rating scale were selected. Of the 5 sCAA patients 1 year follow-up scans were available. Both FOCAS and AURORA are performed in accordance with the declaration of Helsinki. Informed consent was obtained from all participants and study protocols were approved by the local IRB.

All participants were scanned on a 3.0 Tesla MRI scanner (Philips Achieva, Best, the Netherlands) and data was acquired using a standard 32-channel head coil. The protocol included SWI acquired with the following parameters: 4 echoes, TR/first TE/echospacing of 31/7.2/6.2 ms, flip angle 17 degrees, 130 slices and an FOV of 230 × 190 × 130 mm with a voxel size of 0.6 × 0.6 × 1 mm resulting in a scan duration of 3:31 min. cSS was assessed using the multifocality scale as described in Charidimou et al. (2017)*.* In short: each hemisphere was scored separately with a score of 0–2; where 0 represents no cSS present, 1 represents one sulcus or up to three immediately adjacent sulci with cSS and 2 represents two or more non– adjacent sulci or more than 3 adjacent sulci with cSS. Scores of both hemispheres are added to get a 0–4 scale. cSS potentially connected to lobar ICH were not included in this rating.

### 2.1 Development and performance of the cSS tool

SWI-images were processed using a custom pipeline created in MeVisLab 3.2 (Bremen, Germany). Images were preprocessed with a 2D-vesselness filter using a single scale with a sigma of one voxel (Frangi et al., 1998). In short, the vesselness filter is based upon the Hessian matrix with the output showing how similar the local neighborhood is to a hypointense tubular structure. The resulting segmentation was then visually evaluated and seed points for a 3D-6-Neighborhood (x,y,z) growing region algorithm were placed within a cSS region. The resulting mask was again visually inspected, corrected for any false positives and the volume of cSS was calculated. This semi-automatic segmentation process was executed by an experienced rater (E.K., 5 years of experience in the field), blind to clinical data, on two separate occasions within a four-month period, to assess intra-observer agreement. The code for this tool will be made available upon reasonable request. Reproducibility between these sessions was determined by calculating the Pearson’s correlation coefficient on the resulting cSS volume using SPSS software version 25.0 (*IBM*, Chicago, IL, USA). Agreement was also plotted in an R^2^ correlation plot and a Bland-Altman plot in Matlab 2016a (*Mathworks*, Natick, MA, USA). Furthermore, to assess inter-observer agreement, a second experienced rater (S.V., 5 years of experience in the field) rated the same dataset as the first rater, blind to clinical data and the outcome of the assessment by the first rater. The two raters were compared with an absolute agreement intraclass correlation coefficient (ICC) on the resulting cSS volume using SPSS software version 25.0 (*IBM*, Chicago, IL, USA). The second rater was trained on example data of three patients (not included in the final evaluation dataset) while providing the output of the first rater as ground truth.

To assess to what extent segmentations were in exact agreement on the voxel-level, the Dice similarity coefficient (DSC) (see Eq. 1: area of overlapping voxels divided by the sum of the areas of both ROIs) (Bonar et al., 1993) was calculated:

(1) $$\mathrm{DSC}=\;\frac{\left(2*(\left|{\mathrm{ROI}}_{\mathrm{observation}\;1}\right|\;\cap \left|{\mathrm{ROI}}_{observation2}\right|\right)}{\left|{\mathrm{ROI}}_{observation1}\right|+\left|{\mathrm{ROI}}_{observation2}\right|}$$

Finally, the volumes of cSS measured with the newly developed tool were plotted against the standard cSS multifocality rating scale.

To assess the effectiveness of such a quantitative marker for follow-up research, the 1-year follow-up scans of the sCAA patients were processed by the same experienced rater (E.K.), blind to any clinical data. These volumes were compared to the baseline volumes and to a previous developed visual progression scale that has been derived and validated to be sensitive to change over time and predictive of future ICH (Pongpitakmetha et al., 2020).

## 3 Results

Mean age of the patients was 60 ± 13 years (54 ± 9 years for D-CAA and 79 ± 4 years for sCAA) and 35% were women. Fourteen of the 20 patients had a history of ICH and three a history of TFNEs.

**Fig. 1:**
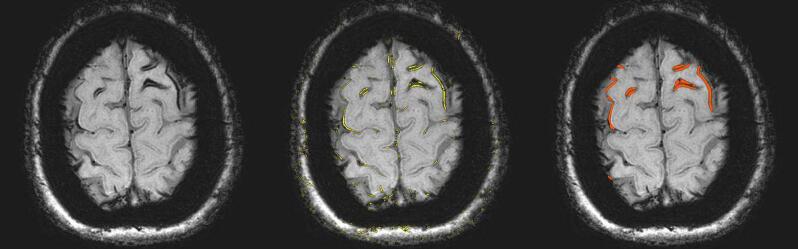
Susceptibility weighted scan of a patient with CAA showing cSS in multiple sulci in both hemispheres. On the left panel the unprocessed image is shown. On the middle panel, the image processed with a 2D-vesselness filter is shown with voxels in yellow that have an above threshold value of vesselness, i.e. similarity to a hypointense tubular structure. In this initial segmentation,. many false positives can be observed. Subsequently to this initial segmentation seed-points were placed followed by a 3D region-growing algorithm. On the right panel, the resulting segmentation is shown in red highlighted areas. These are then used for further analysis. (For interpretation of the references to colour in this figure legend, the reader is referred to the web version of this article.)

**Fig. 2:**
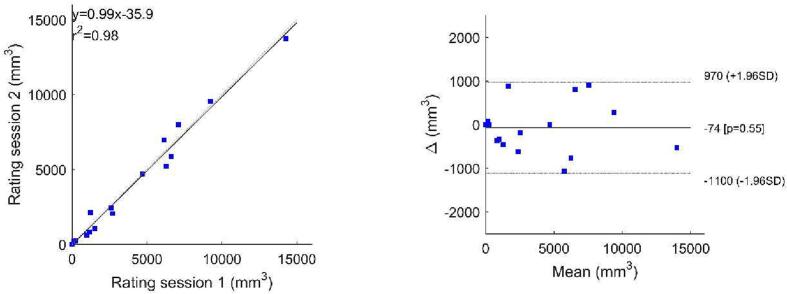
Correlation and Bland Altman plot for volumes recorded in rating session 1 versus rating session 2. The correlation plot shows good agreement and in the Bland Altman plot no large outliers or trends are observed between the two scoring sessions.

**Fig. 3:**
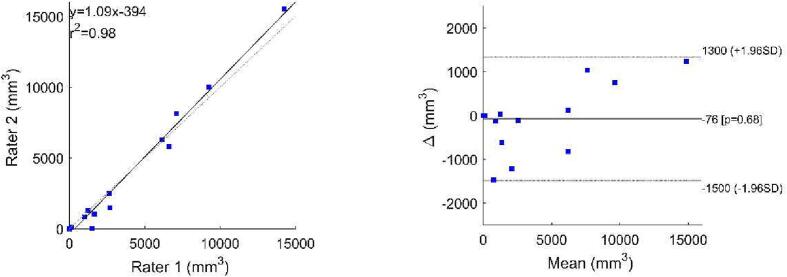
Correlation and Bland Altman plot for volumes recorded by rater 1 versus rater 2. The correlation plot shows good agreement and in the Bland Altman plot no large outliers or trends are observed between the two raters.

Processing of the SWI files by use of the 2D-vesselness filter resulted in a suppression of all non-tubular shapes; as shown on the white overlay in the middle pane of Fig. 1. This middle pane shows that application of this vesselness filter allows segmentation of all cSS, but also of several other hypointense tubular structures such as some veins. A slice of the same patient, processed by the vesselness filter, at the height of the Sylvian fissure is shown in Supplementary data, only false positives are seen in this figure. Placement of seed points within the cSS and execution of the growing region algorithm resulted in a good segmentation of the cSS lesions with the resulting mask shown on the right in Fig. 1. Intra-observer agreement was excellent for cSS volume (Pearson’s 0.991, P < 0.001), high for r^2^ (0.98) and agreement was good in the Bland Altman-plot (Fig. 2). Also, inter observer agreement was excellent with an ICC of 0.995 (P < 0.001, 95 %CI: 0.984–0.998), a high r^2^ (0.98) and good agreement in the Bland Altman-plot (Fig. 3).

While total cSS volume was highly reproducible, the voxels included in segmentations on separate occasions were not always the same (mean DSC 0.75 ± 0.15); we consider this DSC values a reflection of good agreement, due to the sparseness and small sizes of the segmented areas.

**Fig. 4:**
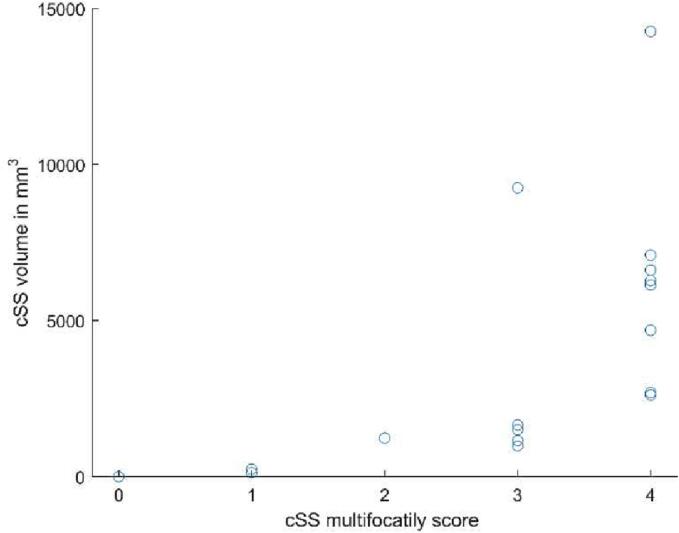
Volume of cortical superficial siderosis plotted against the multifocality rating score; in the highest multifocality rating scale categories a large spread in cSS volume is demonstrated, suggesting that in patients with severe cSS, this segmentation and quantification method may prove to be a valuable tool in tracking progression.

According to the cSS multifocality rating scale, two of the 20 participants were classified as score 1, one as 2, five as 3 and eight as 4. The relationship between this categorical classification and the quantified cSS volumes is shown in Fig. 4. The eight participants who were classified as having the most severe cSS score (score 4) showed a wide range of quantified cSS volumes, ranging from 2.7 mL to 14.3 mL. Also, one individual showed a high quantified cSS volume despite being classified in the third category, because of extensive cSS mainly localized in the right hemisphere (adding two points to the categorical score), while the left hemisphere only had a minor quantity of cSS (adding only one additional point).

**Fig. 5:**
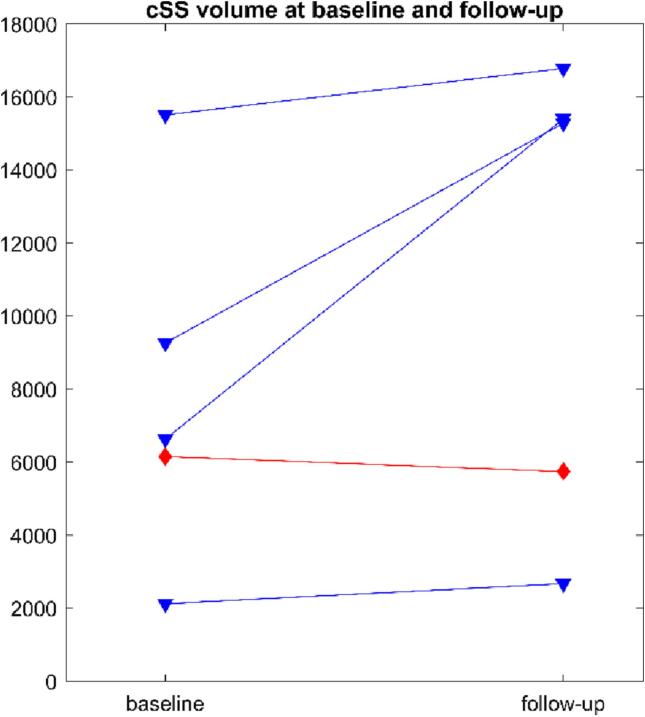
Volumes of cSS segmentation at baseline and follow-up of the 5 sCAA patients. Blue triangles indicate patients in whom an increase in cSS was found as described by the progression scale, red diamonds indicate subjects in whom no cSS increase was reported. A marked increase in volume is found only for those patients in whom the neuroradiologist reported an increase in cSS. (For interpretation of the references to colour in this figure legend, the reader is referred to the web version of this article.)

Results of segmentation of baseline and follow-up scans are shown in Fig. 5: increase in volume as an increase in cSS was found as described the progression scale (Pongpitakmetha et al., 2020) shown in blue, whereas scans absent of this observation are shown in red. Two of the patients who showed an increase in cSS were already in category 4. of the multifocality scale (Charidimou et al., 2017).

## 4 Discussion

Our proof-of-principle study demonstrates in a group of patients with CAA with varying cSS severity that quantification of cSS volume can be achieved in a reproducible way by a relatively simple, semi-automatic tool based upon region growing of vesselness-enhanced SWI images. Clear advantages of cSS volume quantification as compared to a categorical scale are twofold: it does not exhibit a ceiling effect and it can also identify minor progression. Quantification of cSS volume is important as many patients with advanced CAA readily fall into the highest category of the multifocality rating scale; further progression of cSS in these patients cannot be reflected in this scale and will therefore remain unnoticed. Also, our study showed that in patients with the highest score on the categorical scale a wide variability of cSS volumes was present, suggesting that subjects in this category are more heterogenous than implied by their similar cSS score. Furthermore, in the two patients in whom our neuroradiologist identified an increase in cSS on the 1-year follow-up scans, our tool also identified an increase in volume, even while the categorical scale remained unchanged since these subjects were already in the highest category. Whether the use of our quantitative score will show that these subjects also differ with respect to clinical disease progression needs to be studied in future research.

The semi-automatic approach enables quantification of cSS in an accurate manner and can be applied accurately by experienced raters with good agreement: usage of this method showed excellent intra- and inter-observer reliability. Our data suggests that the automatic segmentation using a 2D-vesselness filter combined with a 3D growing region algorithm, with elimination of false positives by a human rater, yields sufficient quality to be used in further clinical validation studies.

While this method of quantification seems highly relevant in cSS related research, the added value in clinical practice is hard to assess. This caveat is similar to the multifocality scale which also only finds its place in a research setting. Furthermore, as a proof of principle paper we did not include any correlations with clinical data or outcomes, and therefore it is unclear if the extra time spent making segmentations is warranted and feasible for clinical practice.

A weakness of MRI is that the hypointense area caused by cSS is dependent on field strength, sequence-parameters and post-processing approaches (Nandigam et al., 2009, Greenberg, 2009). Therefore, our quantitative score may be difficult to compare between centers and it would be important to standardize imaging parameters and post-processing. Luckily, many patients will be followed-up in a single center, making it easier to guarantee consistent identification of cSS volume change. To increase comparability of scans obtained with different settings, one could envision calibration scales based upon MR-physics theory predicting the relative amplification of the hypointense area due to the presence of hemosiderin. However, magnetic susceptibility characteristics of hemosiderin are variable and the influence on the magnetic field distribution will be shape and orientation dependent (Salomir et al., 2003), ultimately limiting the performance of such recalibration efforts. Alternatively, repeated in vivo scanning sessions could be used for such a calibration scale, or measurements on dedicated phantoms. Another option would be to use internal calibration measures, such as percentage of total sulci length affected. Such an internally calibrated measure could be an improvement over volume, since the influence of sequence parameters and field strengths will be more pronounced in the perpendicular, than in the longitudinal direction of the sulcus. Furthermore, measures like surface area of the cortex affected may prove to be a more useful or accurate measure than volume. However, this will almost certainly lead to an increase in processing time and the chance of errors in segmentation of the sulci and cortical surface, especially in a population with many lobar bleeds. From our largely cross-sectional dataset it is not possible to determine which of these measures would perform best with respect to clinical outcome and/or as a measure of disease progression. However, the cSS segmentations of our method, prior to quantification of the volume, can be used as input for more advanced processing and subsequently provide more useful outcome measures, like surface area. As our study was conducted on a single center 3 T system, this impact has to be investigated in other studies, for example in multi-center studies on CAA progression.

Our study has a relatively small sample size. However, despite this small sample size we are able to show a large heterogeneity in the higher categories of the multifocality scale, and we are able to show that our novel method can indeed identify progression. A more accurate assessment of cSS progression might improve prediction of future ICH which is of clinical importance for example to better inform patients about prognosis or in case patients are on antiplatelet therapy or on anticoagulation. Also, for research purposes, a quantitative score might be preferable for example when progression of cSS is used as a biomarker. Future studies are needed to investigate the clinical relevance of our new scale.

Another limitation of this method is that it can be more time consuming than applying a multifocality scale: in the high resolution images produced in this MRI protocol processing may take up to an hour in patients with extensive cSS. Furthermore, we also did not assess the scan-rescan variability of the method. However, due to the high quality and resolution of the scans used in our protocol we expect the scan-rescan variability to be less important than the inter-observer variability. Should this method be applied to data with a lower resolution, planning and partial voluming effects will start to have an impact on the final quantification. Also, no one-to-one comparison was made between manual segmentations and cSS-segmentations based on our semi-automatic approach. However, because both methods are observer-based, little deviations should be expected.Future studies will also have to elucidate the association between quantified cSS volume and other MRI markers of CAA pathology, together with its prognostic value for future ICH risk in clinical practice. The availability of cSS masks also present the possibility of further research with respect to localized clinical features (i.e. distance of other pathological processes to cSS), as well as even more detailed analyses of cSS (i.e. shape features). Furthermore, longitudinal studies performed in similar scanning conditions potentially can further show how cSS progresses in the course of CAA. Lastly, the additional *clinical* value of quantification of cSS compared with the categorical classification for prognosis has to be investigated.

In conclusion, semi-automatic segmentation and quantification of cSS on SWI-MRI is feasible and reproducible and may be used as a more continuous variable of cSS severity, although it should only be used on images acquired under similar conditions.

## CRediT authorship contribution statement

**T.W. van Harten:** Conceptualization, Formal analysis, Investigation, Methodology, Resources, Software, Validation, Visualization, Writing – original draft. **E.A. Koemans:** Conceptualization, Formal analysis, Investigation, Methodology, Resources, Validation, Writing – review & editing. **S. Voigt:** Formal analysis, Investigation, Methodology, Resources, Validation, Writing – review & editing. **I. Rasing:** Resources, Writing – review & editing. **M.J.P. van Osch:** Conceptualization, Funding acquisition, Investigation, Methodology, Supervision, Writing – review & editing. **M.A.A. van Walderveen:** Conceptualization, Investigation, Supervision, Writing – review & editing. **M.J.H. Wermer:** Conceptualization, Funding acquisition, Supervision, Writing – review & editing.

## Declaration of Competing Interest

The authors declare that they have no known competing financial interests or personal relationships that could have appeared to influence the work reported in this paper.

## Data Availability

Data will be made available on request.

## References

[b0005] Bonar D.C., Schaper K.A., Anderson J.R., Rottenberg D.A., Strother S.C. (1993). Graphical analysis of MR feature space for measurement of CSF, gray-matter, and white-matter volumes. J. Comput. Assist. Tomogr..

[b0010] Charidimou A. (2017). Emerging concepts in sporadic cerebral amyloid angiopathy. Brain.

[b0015] Charidimou A. (2022). The Boston criteria version 2.0 for cerebral amyloid angiopathy: a multicentre, retrospective, MRI-neuropathology diagnostic accuracy study. Lancet Neurol..

[b0020] Charidimou A., Boulouis G., Xiong L.i., Jessel M.J., Roongpiboonsopit D., Ayres A., Schwab K.M., Rosand J., Gurol M.E., Greenberg S.M., Viswanathan A. (2017). Cortical superficial siderosis and first-ever cerebral hemorrhage in cerebral amyloid angiopathy. Neurology.

[b0025] Charidimou A., Boulouis G., Roongpiboonsopit D., Auriel E., Pasi M., Haley K., van Etten E.S., Martinez-Ramirez S., Ayres A., Vashkevich A., Schwab K.M., Goldstein J.N., Rosand J., Viswanathan A., Greenberg S.M., Gurol M.E. (2017). Cortical superficial siderosis multifocality in cerebral amyloid angiopathy: A prospective study. Neurology.

[b0030] Charidimou A., Boulouis G., Xiong L.i., Pasi M., Roongpiboonsopit D., Ayres A., Schwab K.M., Rosand J., Gurol M.E., Viswanathan A., Greenberg S.M. (2019). Cortical Superficial Siderosis Evolution. Stroke.

[b0035] Frangi A.F. (1998).

[b0040] Greenberg S.M. (1993). The clinical spectrum of cerebral amyloid angiopathy: presentations without lobar hemorrhage. Neurology.

[b0045] Greenberg S.M. (2009). Cerebral microbleeds: a guide to detection and interpretation. Lancet Neurol..

[b0050] Greenberg S.M., Smith E.E. (2019). Implications of cortical superficial siderosis in CAA. Superficial relationships.

[b0055] Jäkel L., De Kort A.M., Klijn C.J.M., Schreuder F.H.B.M., Verbeek M.M. (2022). Prevalence of cerebral amyloid angiopathy: A systematic review and meta-analysis. Alzheimers Dement..

[b0060] Knudsen K.A., Rosand J., Karluk D., Greenberg S.M. (2001). Clinical diagnosis of cerebral amyloid angiopathy: validation of the Boston criteria. Neurology.

[b0065] Linn J., Halpin A., Demaerel P., Ruhland J., Giese A.D., Dichgans M., van Buchem M.A., Bruckmann H., Greenberg S.M. (2010). Prevalence of superficial siderosis in patients with cerebral amyloid angiopathy. Neurology.

[b0070] Nandigam R.N.K., Viswanathan A., Delgado P., Skehan M.E., Smith E.E., Rosand J., Greenberg S.M., Dickerson B.C. (2009). MR imaging detection of cerebral microbleeds: effect of susceptibility-weighted imaging, section thickness, and field strength. AJNR Am. J. Neuroradiol..

[b0075] Pongpitakmetha T. (2020). Cortical superficial siderosis progression in cerebral amyloid angiopathy: Prospective MRI study. Neurology.

[b0080] Salomir, R., B.D. de Senneville, and C.T. Moonen, A fast calculation method for magnetic field inhomogeneity due to an arbitrary distribution of bulk susceptibility. 2003. **19B**(1): p. 26-34.

[b0085] Vinters H.V. (1987). Cerebral amyloid angiopathy. A critical review. Stroke.

[b0090] Wermer M.J.H., Greenberg S.M. (2018). The growing clinical spectrum of cerebral amyloid angiopathy. Curr. Opin. Neurol..

