# Supplementary material for: Quantitative measurement of cortical superficial siderosis in cerebral amyloid angiopathy
Source: Neuroimage Clin. 2023 Jun 1;38:103447. doi: 10.1016/j.nicl.2023.103447 (PMC10258504; doi:10.1016/j.nicl.2023.103447)
Supplement: Supplementary Data 1 [file mmc1.docx]

**Supplementary Figures:**


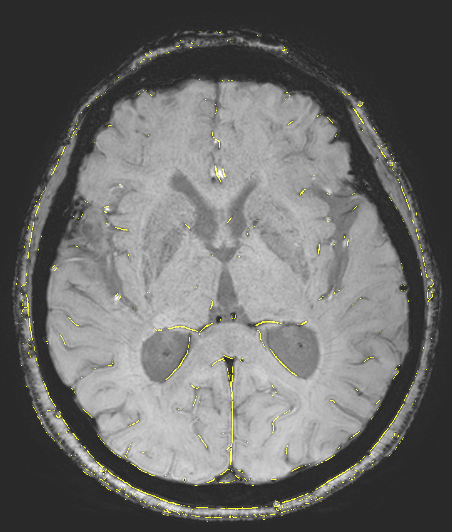


Supplementary figure 1: A slice of the SWI scan at the height of the Sylvian fissure of the same patient as shown in Figure 1. Some false positives are segmented in the initial segmentation by vesselness filter. These are excluded by the following step with the seed region growing algorithm.
